# Constitutive apoptosis in equine peripheral blood neutrophils in vitro

**DOI:** 10.1016/j.tvjl.2014.08.029

**Published:** 2014-12

**Authors:** Timothy J. Brazil, Padraic M. Dixon, Christopher Haslett, Joanna Murray, Bruce C. McGorum

**Affiliations:** a Royal (Dick) School of Veterinary Studies and the Roslin Institute, University of Edinburgh, Roslin, Midlothian EH25 9RG, UK; b Queen's Medical Research Institute (QMRI), Medical School, University of Edinburgh, Edinburgh EH16 4TJ, UK; c Rayne Laboratory, Medical School, University of Edinburgh, Teviot Place, Edinburgh EH8 9AG, UK

**Keywords:** Equine, Polymorphonuclear leucocyte, Neutrophil, Apoptosis

## Abstract

The aim of this study was to characterise constitutive apoptosis in equine peripheral blood neutrophils, including assessment of factors that potentially modulate neutrophil survival through alteration of the rate of constitutive apoptosis. Cells underwent spontaneous time-dependent constitutive apoptosis when aged in culture for up to 36 h, developing the structural and functional features of apoptosis observed in many cell types, including human neutrophils. Neutrophils undergoing apoptosis also had diminished zymosan activated serum (ZAS)-stimulated chemiluminescence, but maintained responsiveness to phorbol myristate acetate (PMA). The constitutive rate of equine neutrophil apoptosis was promoted by lipopolysaccharide (LPS), tumour necrosis factor α and phagocytosis of opsonised ovine erythrocytes, while it was inhibited by dexamethasone and ZAS (a source of C5a). Formyl-Met-Leu-Phe, leukotriene B_4_, platelet activating factor and PMA had no demonstrable effect on equine neutrophil apoptosis. There was a difference between equine and human neutrophil apoptosis in response to LPS and the time-dependence of the response to dexamethasone.

## Highlights

• Equine peripheral blood neutrophils underwent spontaneous time-dependent constitutive apoptosis when aged in culture for up to 36 h.
• Equine neutrophils undergoing apoptosis develop the structural and functional features of apoptosis observed in many cell types, including human neutrophils.
• Neutrophils undergoing apoptosis had diminished zymosan activated serum-stimulated chemiluminescence, but maintained responsiveness to phorbol myristate acetate.
• The constitutive rate of neutrophil apoptosis was promoted by lipopolysaccharide, tumour necrosis factor α and phagocytosis of opsonised ovine erythrocytes, while it was inhibited by dexamethasone and ZAS (a source of C5a).
• Equine and human neutrophil apoptosis differed in terms of responses to lipopolysaccharide and in the time-dependence of the response to dexamethasone.

## Introduction

Equine neutrophils play a key role in host defence, but also contribute to host tissue injury through secretion of pro-inflammatory and histotoxic agents (Morris, 1991; Moore et al., 1995; de la Rebière de Pouyade and Serteyn, 2011). This has prompted investigation into the role of neutrophil apoptosis in equine pulmonary (Turlej et al., 2001; Brazil et al., 2005; Lavoie-Lamoureux et al., 2012), gastrointestinal (Krista, 2012) and orthopaedic (Kim et al., 2003) diseases. Neutrophils are programmed to undergo constitutive apoptosis, which, in contrast to necrosis, locks the cell into a non-secretory mode and initiates rapid engulfment and removal by phagocytes without inducing an inflammatory response, thereby limiting host tissue injury (Mecklenburgh et al., 1999).

The aims of this study were to characterise constitutive apoptosis of equine peripheral blood neutrophils when these cells were aged in culture for up to 36 h and to determine the effects of various stimuli on the rate of constitutive apoptosis in vitro. Stimuli used were phorbol myristate acetate (PMA), zymosan activated serum (ZAS, a source of C5a), the bacterial product formyl-Met-Leu-Phe (fMLP), leukotriene B_4_ (LTB_4_), platelet activating factor (PAF), tumour necrosis factor α (TNF-α), lipopolysaccharide (LPS), dexamethasone (DEX) and phagocytosis of opsonised ovine erythrocytes.

## Materials and methods

### Isolation and culture of peripheral blood neutrophils

Venous blood was collected from clinically healthy horses into 1:10 V/V 3.8% sodium citrate (Phoenix Pharmaceuticals), with ethical approval granted under UK Home Office Licence. For some experiments, potential inter-species differences were investigated by parallel examination of human peripheral blood neutrophils. Blood was collected from healthy human volunteers with ethical approval (Lothian Health 1702/95/3/11; date of approval 9 August 1995). Fresh peripheral blood neutrophils were isolated using discontinuous plasma/Percoll gradients (Brazil et al., 1998), suspended at 5 × 10^6^/mL in Iscove's modified Dulbecco's medium (Gibco) supplemented with 10% autologous serum, 100 U/mL penicillin (Sigma) and 100 µg/mL streptomycin (Sigma), and aged by culturing for up to 36 h in flat-bottomed polypropylene flexiwells (Becton Dickinson) at 37 °C in a humidified 5% CO_2_ atmosphere (Brazil et al., 2005).

### Morphology

**Fig. 1:**
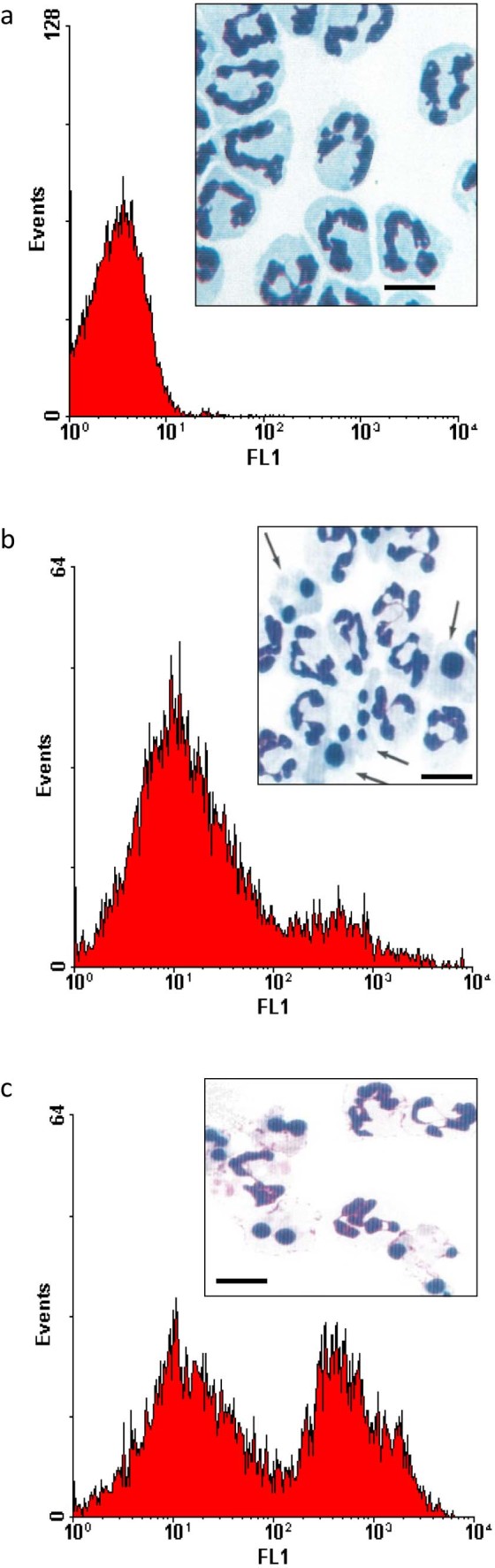
Annexin V binding (10,000 events) and light microscopic morphology (Diff-Quik, insert panels) of equine peripheral blood neutrophils aged in culture for (a) 0, (b) 8 and (c) 20 h. Arrows indicate cells with apoptotic morphology. Scale bar = 10 µM.

After culture, apoptosis was assessed by light and transmission electron microscopy (Savill et al., 1989; Brazil et al., 2005). For light microscopy, apoptotic neutrophils were defined as cells containing one or more darkly staining pycnotic nuclear remnants (Fig. 1).

### Chromatin fragmentation

DNA of cells undergoing apoptosis fragments in a characteristic internucleosomal pattern that is recognised as a ‘ladder’ pattern on electrophoresis of DNA. Neutrophils (4 × 10^6^) were aged in culture, harvested, centrifuged at 2000 *g* for 2 min and lysed in 0.5 mL lysing solution containing 6 M guanidine hydrochloride, 20 mM Tris pH 8.0 and 0.1% N-lauryl sarcosine (Sigma). Tris-Cl pH 8.0-buffered phenol/CHCl_3_ (1:1) was added and the organic phenol/CHCl_3_ phase was separated by centrifugation at 11,300 *g* for 10 min. The upper DNA-containing phase was removed and precipitated by adding 1:1 V/V propan-2-ol and 1:10 V/V 3 M sodium acetate. DNA was separated by centrifugation at 11,300 *g* for 5 min and dissolved in a 100 µL solution containing 10 mM Tris-Cl, 1 mM EDTA-Na (pH 8.0) and 50 µg/mL RNase A (Sigma). After incubation at 37 °C for 15 min, DNA was separated by electrophoresis on 1.6% agarose gels at 100 V for 2 h through 0.5× buffer containing 89 mM Tris, 89 mM boric acid and 5 mM ethylene diamine tetraacetic acid (EDTA). Gels were stained with 0.5 µg/mL ethidium bromide and photographed over an ultraviolet (UV) transilluminator. A 1 kilobase pair DNA ladder marker (Life Technologies) was included.

### Annexin V binding

Exposure of phosphatidylserine on outer membrane leaflets of cells undergoing apoptosis can be detected by annexin V binding (Homburg et al., 1995). Neutrophils were harvested and incubated on ice for 10 min with fluorescein isothiocyanate (FITC)-labelled annexin V diluted 1:4 in annexin V buffer (Biowhittaker) and 10,000 events were analysed by FACScan flow cytometry (Becton Dickinson).

### In situ identification of chromatin condensation and DNA fragmentation

To facilitate simultaneous identification of chromatin condensation and DNA fragmentation in cytospin preparations of apoptotic neutrophils, terminal 3'-OH groups of fragmented DNA were labelled in situ by terminal deoxynucleotide transferase (Tdt)-mediated UTP nick end-labelling (TUNEL, FragEL DNA Fragmentation Detection Kit, Calbiochem; Brazil et al., 2005).

### Receptor-mediated respiratory burst activity

Freshly isolated neutrophils mount a respiratory burst in response to ZAS and PMA (Brazil et al., 1998). To investigate whether this capacity was maintained as cells underwent apoptosis and whether different mechanisms of cellular activation (receptor-mediated through ZAS vs. direct activation of protein kinase C by PMA) were important, superoxide anion generation in response to ZAS (10% V/V; Sigma) and PMA (100 ng/mL; Sigma) was assessed using lucigenin dependent chemiluminescence (LDCL) before and after 20 h in culture (Brazil et al., 1998).

### Constitutive apoptosis of equine and human neutrophils

Equine and human peripheral blood neutrophils (625,000 cells/135 µL) were aged in culture for up to 36 h with either medium (control) or test agent (15 µL), namely LPS from *Escherischia coli* serotype 0111:B4 (Sigma; 0.1 ng/mL-10 µg/mL; from 1 mg/mL stock in phosphate buffered saline, PBS; disaggregated by sonication), PAF (Sigma; 1 µM; from 10 mM stock in ethanol), fMLP (Sigma; 1 µM; dissolved in dimethyl sulphoxide, DMSO, then diluted to 1 mM in PBS), PMA (Sigma; 0.1–10 ng/mL; dissolved in DMSO then diluted to 1 mM in PBS), LTB_4_ (kindly donated by Professor A. Rossi, University of Edinburgh, UK; 100 nM; from 1.49 × 10^−4 ^M stock in ethanol), equine recombinant TNF-α (erTNF-α, kindly donated by Dr M. Barton, University of Georgia, USA; 0.1 pg/mL-1 ng/mL in PBS), DEX (Sigma, 0.1 nM-1 µM; from 8.33 mM stock solution in Iscove's modified Dulbecco's medium; Gibco) or 10% V/V ZAS.

To investigate the effect of LPS on neutrophil apoptosis, neutrophils were aged in the presence of other forms of LPS (a different batch of *Escherichia coli* O111.B4 and rough mutant *Salmonella enterica* serovar Typhimurium LPS Ra 60 (kindly supplied by Professor I. Poxton, University of Edinburgh, UK). In additional, the effects of equine TNF-α neutralising antibody (kindly donated by Professor R. MacKay, University of Florida, USA) or control murine isotype-matched monoclonal antibody (MAB002, R&D Systems) on the pro-apoptotic effect of LPS was determined. Apoptosis was assessed by observation of light microscopic features and confirmed by DNA fragmentation studies.

### Effects of phagocytosis on the rate of constitutive equine neutrophil apoptosis

Equine neutrophils were cultured with opsonised ovine erythrocytes (OsRBC) or control non-opsonised ovine erythrocytes (sRBC) at a ratio of 1:3. OsRBC were prepared freshly by centrifuging (800 *g*, 10 min) 10 mL ovine blood containing 3.8% sodium citrate, harvesting and washing 2 mL erythrocyte pellet three times in PBS, resuspending in PBS to a haematocrit of 1% and incubating (37 °C, 30 min) with a sub-aggregating concentration (1/40) of canine anti-ovine erythrocyte antibody (VMRD). Cells were washed twice, then resuspended in Iscove's modified Dulbecco's medium. Control sRBC were prepared identically, except with PBS incubation alone. Proportions of apoptotic and phagocytic neutrophils were determined by light microscopy.

### Statistical analysis

Data were analysed using analysis of variance (ANOVA), followed by the Student–Newman–Keul's post-test or the Student's paired *t* test. Correlations were examined using Spearman's rank sign test. Results are expressed as means ± standard errors of the means (SEM) for *n* sets of separate experiments and were considered to be significant at *P *<* *0.05.

## Results

### Time-dependent constitutive apoptosis of neutrophils

**Fig. 2:**
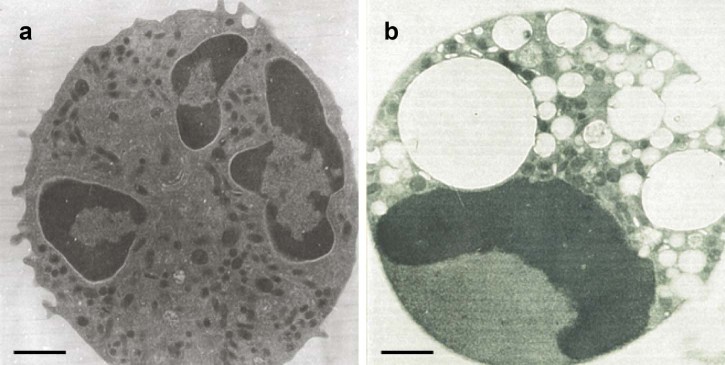
Transmission electron photomicrograph of (a) a freshly isolated equine peripheral blood neutrophil and (b) a neutrophil aged in culture for 20 h. Scale bar = 1 µM.

**Fig. 3:**
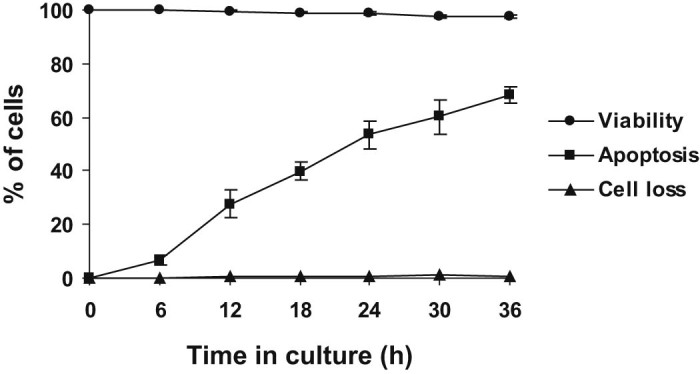
Time course of constitutive apoptosis in equine neutrophils. Cell viability and loss (%) are shown for comparison. Values represent means ± standard errors of the means (SEM) of four separate experiments, each performed in triplicate (apoptosis) or duplicate (viability and cell loss).

Neutrophil viability after 20 h in culture was 99.0 ± 0.4%. Light microscopic features of aged neutrophils were consistent with those of apoptosis in human (Savill et al., 1989; Kerr et al., 1995) and equine (Brazil et al., 2005; Wereszka, 2007) neutrophils. The most prominent features were nuclear pycnosis, with condensation of nuclear chromatin into one or more densely staining, rounded remnants and cytoplasmic vacuolation (Figs. 1b, c), making them readily distinguishable from non-apoptotic neutrophils (Fig. 1a). Ultrastructural examination revealed stereotypical apoptotic morphology (Savill et al., 1989; Kerr et al., 1995); nuclear chromatin was condensed into clearly demarcated, typically ovoid, fragments, with margination of denser, more granular aggregates, which frequently formed a crescent, tightly apposed to the inner nuclear envelope (Fig. 2b). Cytoplasmic vacuolation was prominent and intracellular organelles were retained. Cell membranes remained intact, despite loss of small pseudopodia found in freshly isolated cells (Fig. 2). Nucleolar prominence, a common feature of apoptosis in other cell types (Savill et al., 1989; Kerr et al., 1995) was infrequent. While neutrophils underwent time-dependent constitutive apoptosis, membrane integrity and cell viability were maintained, and cell loss was minimal, despite small but increasing numbers of anucleate cells beyond 24 h (Fig. 3).

### Time-dependent internucleosomal DNA fragmentation of apoptotic neutrophils

**Fig. 4:**
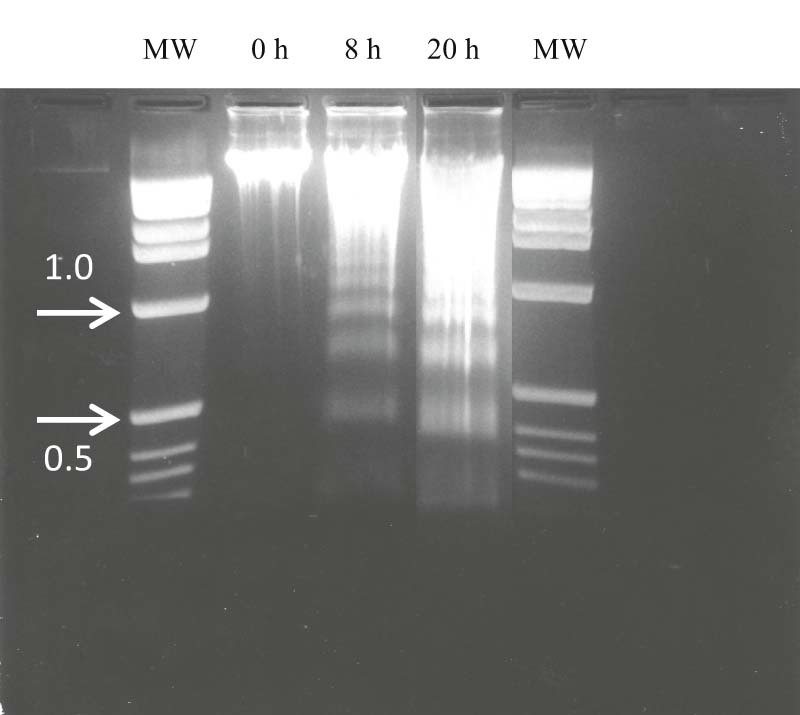
DNA fragmentation in ageing equine neutrophils. Ethidium bromide-stained DNA extracted from freshly isolated neutrophils (0 h) and from cells incubated for 8 and 20 h viewed by ultraviolet fluorescence showing ladder pattern of low molecular weight DNA at 8 and 20 h. The image is representative of three experiments that gave similar results. MW, DNA molecular weight markers; kilobase pairs.

Freshly isolated neutrophils had homogeneous high molecular weight DNA with minimal electrophoretic mobility (Fig. 4). After 8 h in culture, a classical ‘ladder’ pattern of oligonucleosomal DNA fragments was observed; this was enhanced after 20 h (Fig. 4).

### Annexin V binding by apoptotic neutrophils

Freshly isolated neutrophils had minimal annexin V binding, while ageing cells had a time-dependent increase in annexin V binding that paralleled the appearance of apoptotic morphology (Fig. 1). On the basis of morphological criteria, the proportions of neutrophils binding annexin V were strongly correlated with the proportions of apoptotic cells (*r* = 0.92, *P *<* *0.0001).

### In situ identification of chromatin condensation and DNA fragmentation

In cells aged for 8 h, TUNEL labelling clearly localised fragmented DNA to cells with morphologically pycnotic nuclei and demonstrated variation in number and size of condensed chromatin fragments. Negative controls were unlabelled, while positive control samples had widespread, low grade, labelling of morphologically normal cells. Although TUNEL positive cells were readily identified amongst non-apoptotic cells after 8 h in culture, accurate enumeration of TUNEL positive cells at later time points was hampered by non-specific labelling of cells and poor resolution of TUNEL positive cells.

### Receptor-mediated respiratory burst activity

**Fig. 5:**
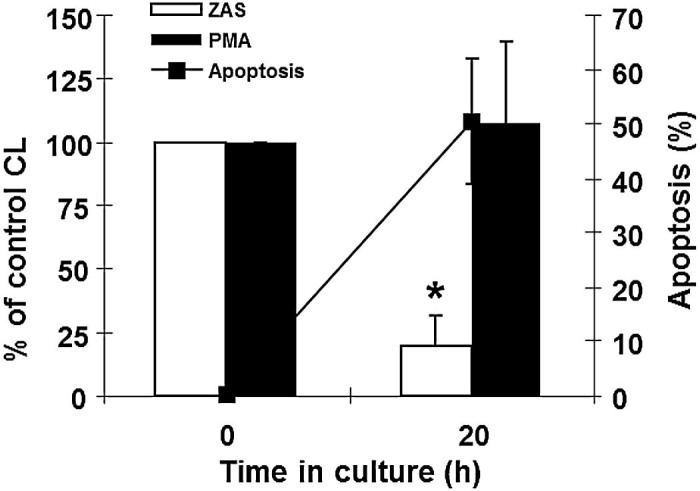
Chemiluminescence response of equine neutrophils undergoing apoptosis. Zymosan activated serum (ZAS; 10% V/V; open bars) stimulated and phorbol myristate acetate (PMA; 100 ng/mL; hatched bars) stimulated lucigenin dependent chemiluminescence (LDCL) was measured in freshly isolated neutrophils and in cells aged for 20 h. Apoptosis (closed squares) was assessed by light microscopy. Chemiluminescence (CL) data represent % of ZAS- and PMA-stimulated LDCL in freshly isolated cells. Values represent means ± standard errors of the means (SEM) of three separate experiments, each performed in triplicate. *P <* *0.05.

After 20 h culture, ZAS-stimulated LDCL significantly decreased to 19.6 ± 12.2% of baseline, despite only 50.3 ± 11.6% cells having morphological features of apoptosis (Fig. 5). This was not due to reduced cell viability, since >99% of cells excluded trypan blue and their PMA stimulated response (107.4 ± 32.3% of baseline) indicated an undiminished capacity to generate a respiratory burst.

### Effects of stimuli on neutrophil apoptosis

**Fig. 6:**
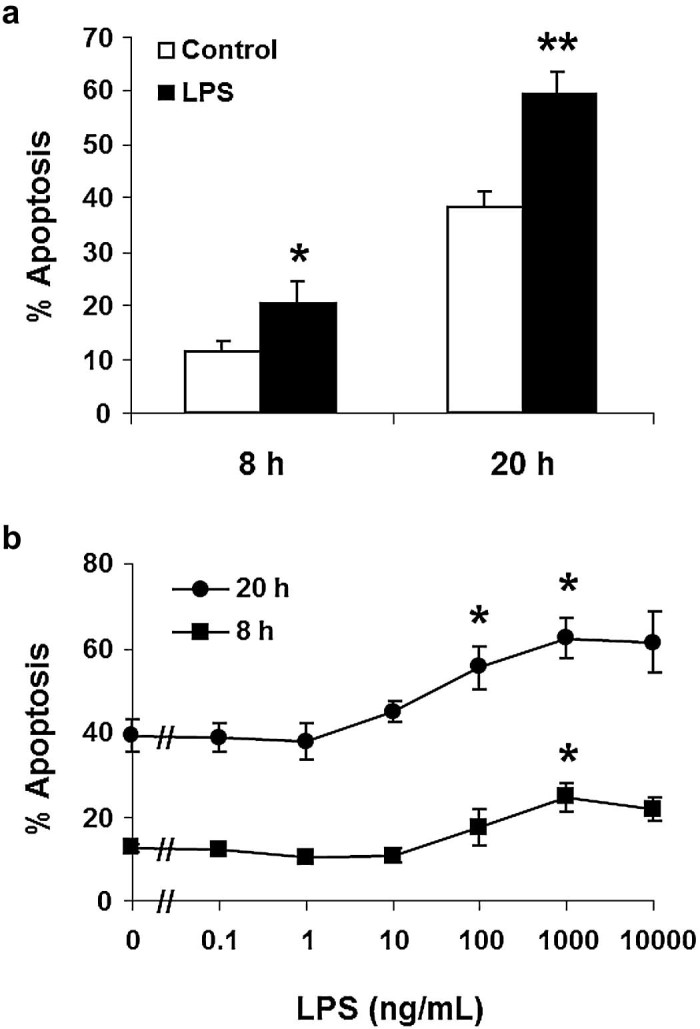
Lipopolysaccharide (LPS) enhances apoptosis of equine neutrophils*.* (a) Cells incubated in the absence (open bars) or presence (closed bars) of 1 µg/mL LPS. Values represent means ± standard errors of the means (SEM) of 10 separate experiments, each performed in triplicate. (b) Cells incubated, either alone or with LPS (0.1–10,000 ng/mL) for 8 h (squares) and 20 h (circles). Values represent means ± SEM of three separate experiments, each performed in triplicate. *P <* *0.05, **P <* *0.001 compared with time-matched control.

ZAS inhibited equine neutrophil apoptosis at 8 h (% apoptosis: control 9.8 ± 2.0%, ZAS 5.6 ± 2.0%; *P *<* *0.05, *n* = 4) and 20 h (control 48.9 ± 3.3%, ZAS 26.7 ± 3.3%; *P *<* *0.001, *n* = 6). Equine rTNF-α caused a small but significant enhancement of apoptosis at 8 h (control 11.2 ± 2.2%, erTNF-α 16.6 ± 3.6%, *P *<* *0.05, *n* = 11) and no significant difference at 20 h (control 43.0 ± 3.1%, erTNF-α 51.3 ± 4.2%, *P* > 0.05, *n* = 11). A series of 10 experiments confirmed that LPS consistently enhanced equine neutrophil apoptosis at 8 and 20 h; the effect was both time dependent (1 µg/mL) and concentration dependent (8 h: EC_50_ 96.7 ± 2.5 ng/mL; 20 h: EC_50_ 32.3 ± 2.4 ng/mL, *n* = 3; Fig. 6). This contrasted with the marked inhibitory effect of LPS on human neutrophil apoptosis at 8 h (control 10.2 ± 1.2 ng/mL, LPS 4.3 ± 1.5 ng/mL; *P *<* *0.05, *n* = 10) and 20 h (control 50.1 ± 8.1 ng/mL, LPS 9.4 ± 1.5 ng/mL; *P *<* *0.05, *n* = 10). Apoptosis was confirmed by DNA fragmentation studies at both 8 and 20 h. Further experiments discounted artefactual causes for this finding. Apoptosis was not attributable to a particular type of LPS, since three forms of LPS consistently promoted apoptosis in equine neutrophils and inhibited apoptosis in human neutrophils at 20 h. Furthermore, equine anti-TNF-α neutralising antibody and control murine isotype-matched monoclonal antibody did not affect basal or LPS-induced apoptosis at 20 h. Neutralising antibody completely abrogated (91.0 ± 2.5%) the priming effect of erTNF-α on fMLP-stimulated chemiluminescence in equine neutrophils, confirming its functional capability, while the isotype-matched control monoclonal antibody had no significant effect. Equine neutrophil apoptosis was not significantly affected by PMA, PAF, fMLP or LTB_4_ (data not shown).

### Effect of dexamethasone on apoptosis of equine neutrophils

**Fig. 7:**
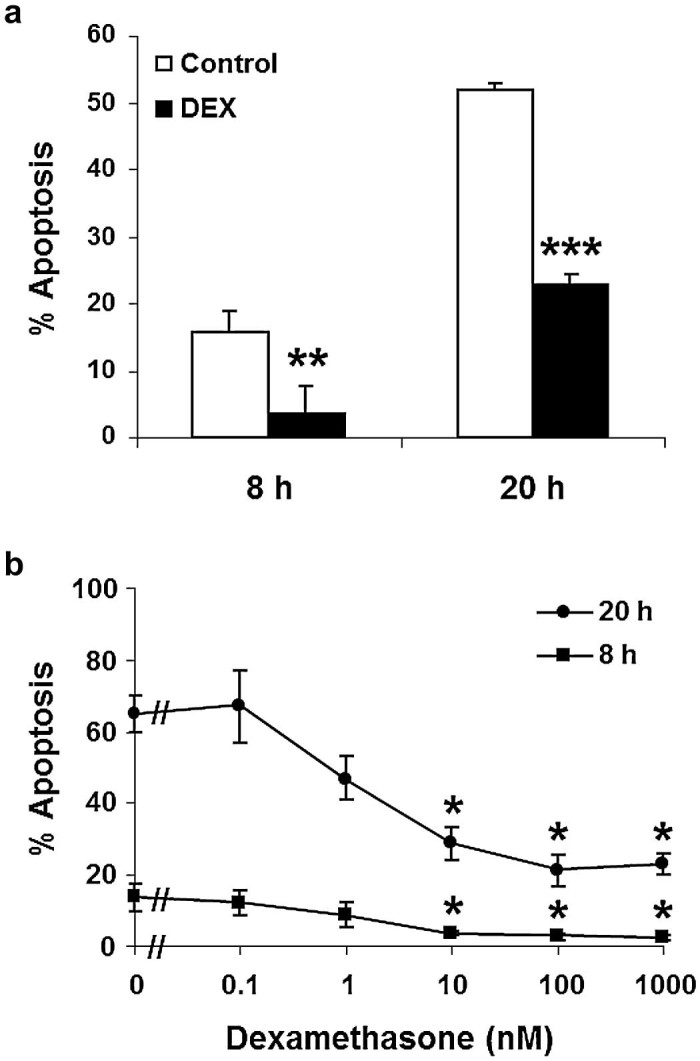
Inhibition of equine neutrophil apoptosis by dexamethasone (DEX). The proportion of apoptotic cells was assessed morphologically. (a) Cells incubated either alone (open bars) or with 1 µM DEX (closed bars). Values represent means ± standard errors of the means (SEM) of eight separate experiments, each performed in triplicate. (b) Cells incubated, either alone or with DEX (0.1–1000 nM), for 8 h (squares) or 20 h (circles). Values represent means ± SEM of three separate experiments, each performed in triplicate. *P <* *0.01, **P <* *0.001, ***P <* *0.0001 compared with time-matched controls.

DEX inhibited equine neutrophil apoptosis at 8 and 20 h (Fig. 7), confirmed by DNA fragmentation studies. This effect was concentration-dependent, with equine neutrophils having similar sensitivity to very low concentrations of DEX (8 h: IC_50_ 0.6 ± 2.6 nM; 20h: IC_50_ 1.1 ± 2.4 nM), similar to that reported for human neutrophils (24 h: IC_50_ 1 nM) by Marwick et al. (2013).

### Effect of phagocytosis on apoptosis of equine neutrophils

**Fig. 8:**
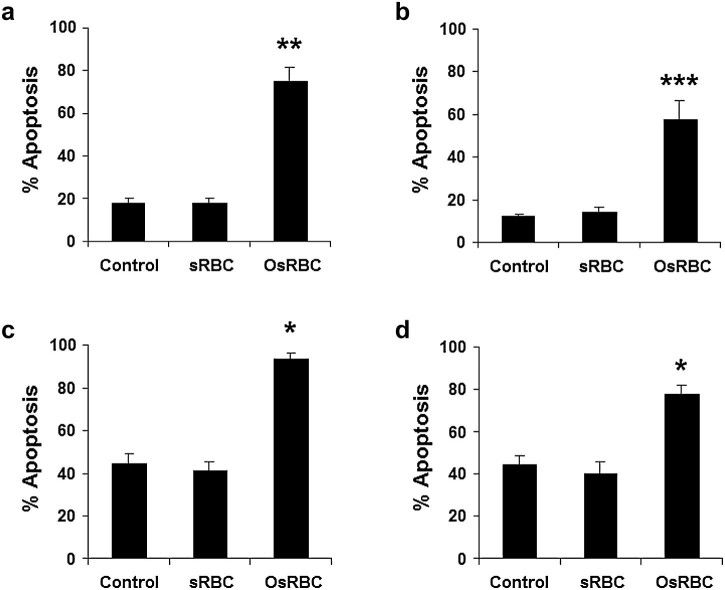
Phagocytosis of opsonised sheep red blood cells (OsRBCs) stimulates apoptosis in equine neutrophils. Neutrophils were incubated for 8 h (a, b) or 20 h (c, d), either in the absence (Control) or presence of non-opsonised (sRBC) or opsonised sheep erythrocytes (OsRBC). (a) Apoptosis assessed morphologically at 8 h. (b) Apoptosis assessed by annexin V binding at 8 h. (c) Apoptosis assessed morphologically at 20 h. (d) Apoptosis assessed by annexin V binding at 20 h. Values represent means ± standard errors of the means of six separate experiments, each performed in triplicate (morphology) or duplicate (annexin V binding). *P <* *0.01, **P <* *0.001, ***P <* *0.0001 compared with time-matched controls.

Incubation with OsRBC markedly and rapidly stimulated equine neutrophil apoptosis (Fig. 8); this effect was statistically significant at 8 h (*P *<* *0.001) and 20 h (*P *<* *0.01), while control sRBC had no significant effect. Apoptosis was confirmed by annexin V binding.

## Discussion

In this study, equine peripheral blood neutrophils underwent spontaneous time-dependent constitutive apoptosis when aged in culture, as demonstrated by light microscopic and ultrastructural features, internucleosomal DNA fragmentation, plasma membrane phospholipid redistribution and maintenance of plasma membrane integrity. These features are recognised in many apoptotic cell types, including human neutrophils (Savill et al., 1989).

Light microscopic features of apoptotic equine neutrophils in this and previous studies (Brazil et al., 2005; Wereszka, 2007), and ultrastructural features noted in the present study, closely resembled those of apoptotic human neutrophils (Savill et al., 1989; Lee et al., 1993). Whilst such ultrastructural alterations remain the gold standard for identification of apoptosis, they must not be considered in isolation. Parallel confirmatory biochemical evidence in this study was obtained from DNA electrophoresis, which demonstrated the classical ‘ladder’ pattern of integer multiples of 180–200 base pair nucleosomes (Wyllie et al., 1980). The TUNEL method of in situ labelling of exposed 3'-OH ends of fragmented DNA (Ben-Sasson et al., 1995) also clearly and specifically identified apoptotic cells in 8 h cultures. Consistent with previous findings (Turlej et al., 2001; Wereszka, 2007; Hirsch et al., 2012; Krista, 2012; Lavoie-Lamoureux et al., 2012), equine neutrophil apoptosis was associated with annexin V binding. Detection of activated caspase-3 has also been employed to assess apoptosis in equine neutrophils (Wereszka, 2007).

The onset of apoptosis is associated with down-regulation of cellular functions. Equine neutrophils had a reduced respiratory burst in response to a receptor-mediated stimulus (ZAS); the magnitude of this reduction (80%) was substantially greater than the proportion of morphologically apoptotic cells (50%). This suggests that cells become functionally isolated from their surroundings at an early stage during apoptosis, prior to developing morphological features of apoptosis. These data concur with findings in human neutrophils, where reductions in both basal (spreading on glass, shape change) and stimulated (shape change, chemotaxis, respiratory burst, degranulation) responses to receptor-mediated agonists (fMLP, C5a) closely mirrored levels of apoptosis in aged cells (Whyte et al., 1993). However, in contrast to the effect of a receptor-mediated stimulus, direct activation of protein kinase C and superoxide anion generation in response to PMA was maintained in aged equine neutrophils. This suggests that signal transduction mechanisms regulating NADPH assembly remain intact in apoptotic cells, but are inaccessible to receptor-mediated stimuli.

The potent anti-apoptotic effect of ZAS (a source of C5a) on equine neutrophils confirmed that inflammatory mediators which prime or activate neutrophil function in vitro may prolong neutrophil survival. Whether the anti-apoptotic effect of ZAS is directly attributable to C5a could be investigated by assessing the effect of human C5a, which is biologically active in equine neutrophils (Rickards et al., 2001). Other inflammatory mediators and bacterial products likely to be active during inflammation in vivo, namely fMLP, LTB4, PMA and PAF, had no demonstrable effect on equine neutrophil survival. Equine rTNF-α caused a small but significant enhancement of apoptosis at 8 h. This contrasts with the anti-apoptotic effect of TNF-α on human neutrophils (Colotta et al., 1992).

The most intriguing and novel finding in this study is the marked pro-apoptotic effect of LPS in equine neutrophils; the concentration dependence of LPS-induced apoptosis (EC_50_ 32.3 ± 2.4 ng/mL at 20 h) is similar to the concentration that primes the respiratory burst (EC_50_ 19.1 ± 4.7 ng/mL; Brazil et al., 1998). The effect was confirmed on multiple occasions using morphological and biochemical criteria. This effect is not a paracrine response secondary to release of TNF-α from neutrophils cultured with LPS (Colotta et al., 1992; Lavoie-Lamoureux et al., 2012); equine TNF-α neutralising antibody and control murine isotype-matched monoclonal antibody did not affect basal or LPS-induced apoptosis at 20 h. This effect is diametrically opposed to the marked concentration dependent inhibitory effect of LPS in human neutrophils noted in this and previous studies (Colotta et al., 1992; Lee et al., 1993; Yamamoto et al., 1993). Assessment of apoptosis by multiple methods in response to a larger panel of LPS types and over a more detailed time course would be required to confirm and further dissect this novel pro-apoptotic response in equine neutrophils.

DEX markedly inhibited apoptosis of equine neutrophils, consistent with previous findings (Hirsch et al., 2012). The sensitivity of equine neutrophils to low concentrations of DEX is comparable to that of human neutrophils (Marwick et al., 2013). While human neutrophils require at least 8 h exposure to DEX before inhibition of apoptosis is evident (Meagher et al., 1996), a significant inhibitory effect on equine neutrophil apoptosis was already apparent at 8 h. The dramatic increase in peripheral neutrophil numbers observed in human beings and horses (Lassen and Swardson, 1995) following systemic administration of glucocorticoids may be partly mediated by inhibition of neutrophil apoptosis and clearance (Meagher et al., 1996). More importantly, this enhanced longevity of DEX-exposed neutrophils in vivo may have implications regarding clearance of these cells from inflammatory sites, which could be detrimental in terms of resolution of inflammation. However, this is balanced by the marked potentiation of phagocytosis in apoptotic neutrophils by glucocorticoid treated macrophages (Liu et al., 1999), suggesting that glucocorticoids may also positively promote resolution of inflammation.

Phagocytosis of opsonised particles, a key host defence function, markedly and rapidly promoted equine neutrophil apoptosis. Since ingestion of non-opsonised sRBC had no such effect, the mechanism of particle recognition and uptake is crucial in determining the effect on apoptosis.

## Conclusions

Equine peripheral blood neutrophils underwent spontaneous time-dependent constitutive apoptosis when aged in culture, developing the structural and functional features of apoptosis observed in many cell types, including human neutrophils. LPS had a consistent pro-apoptotic effect on equine neutrophils which contrasted with its inhibitory effect on apoptosis in human neutrophils, but the mechanism underlying this species difference remains unclear. DEX markedly inhibited constitutive apoptosis of equine neutrophils. The differences between apoptosis of equine and human neutrophils in response to LPS, and the time-dependence of the response to DEX, may be of value in dissecting the underlying regulatory mechanisms of neutrophil apoptosis.

## Conflict of interest statement

None of the authors of this paper has a financial or personal relationship with other people or organisations that could inappropriately influence or bias the content of the paper.
